# First-in-human study to assess the pharmacokinetics, tolerability, and safety of single-dose oxybutynin hydrochloride administered via a microprocessor-controlled intravaginal ring

**DOI:** 10.1080/10717544.2023.2180113

**Published:** 2023-02-22

**Authors:** Willem de Laat, Lisa Pagan, R. Karl Malcolm, Maarten Wiegerinck, Victor Nickolson, Bertine Huisman, Rik Stuurman, Michiel van Esdonk, Naomi Klarenbeek

**Affiliations:** a LiGalli, The Hague, The Netherlands; b Centre for Human Drug Research, Leiden, The Netherlands; c Department of Gynaecology and Obstetrics, Leiden University Medical Centre, Leiden, The Netherlands; d School of Pharmacy, Queen’s University Belfast, United Kingdom; e Department of Clinical Pharmacy and Toxicology, Leiden University Medical Centre, Leiden, The Netherlands

**Keywords:** Vaginal ring, urinary incontinence, *N*-desethyloxybutynin

## Abstract

Polymeric drug-releasing vaginal rings are useful for both local and systemic administration of drugs via the intravaginal route. Typically, they provide continuous sustained or controlled release of drug(s) over extended time periods, thereby avoiding overdose and improving adherence. This first-in-human study (EudraCT number: 2020-0050044-30) evaluated the pharmacokinetics, safety, and tolerability of a single dose of oxybutynin administered by a novel microprocessor-controlled vaginal ring (MedRing). Eight healthy female subjects received an electronically controlled single intravaginal dose of 3 mg oxybutynin hydrochloride (100 mg/mL) dissolved in 1:1 water/propylene glycol administered via MedRing. Following dosing, MedRing was kept *in situ* for up to 6 h. Blood samples were collected 1 h prior to oxybutynin dosing and subsequently at regular intervals post-dose for the assessment of plasma concentrations of oxybutynin and its active metabolite *N*-desethyloxybutynin. The results showed that MedRing efficiently administered oxybutynin via the intravaginal route, resulting in plasma oxybutynin levels comparable to orally administered oxybutynin. The mean ± standard deviation pharmacokinetic parameters for oxybutynin were *C*_max_ 5.4 ± 2.7 ng/mL, AUC_inf_ 34.9 ± 17.4 h ng/mL, *t*_1/2_ 8.5 ± 3.5 h and for N-desethyloxybutynin were *C*_max_ 3.9 ± 2.5 ng/mL, AUC_inf_ 51.1 ± 43.1 h ng/mL, *t*_1/2_ 7.7 ± 5.9 h. No serious adverse events were reported. The study demonstrates that intravaginal administration of oxybutynin hydrochloride using the MedRing device was well tolerated.

## Introduction

The vagina is increasingly used as a route for drug administration (Alexander et al., 2004; Hoffman, 2008; Mathias & Hussain, 2010; Gupta et al., 2011; Adepu and Ramakrishna, 2021; Shewale et al., 2022). The presence of a dense network of blood vessels in the vaginal tissue, its high permeability for low-molecular-weight drugs, and its physiological characteristics make the vagina suitable for systemic drug delivery (Iqbal & Dilnawaz, 2019). In addition, vaginal drug delivery can minimize hepatic first-pass effects and gastrointestinal interferences commonly encountered with oral formulations, which might reduce adverse effects when compared with oral administration (Hussain & Ahsan, 2005; Lopez et al., 2013; López-Picado et al. 2017; Gomaa et al., 2018).

Vaginal rings are a well-established method of vaginal drug delivery, commonly used to deliver contraceptive steroids (either progestogens alone or combinations of progestogen and estrogen), estrogens for postmenopausal hormone replacement, and antiretroviral agents for prevention of human immunodeficiency virus infection (Harwood & Mishell, 2001; Ahrendt et al., 2006; Baeten et al., 2016; Tiboni et al., 2021). However, first-generation vaginal rings – typically in the form of polymeric matrix or reservoir-type devices – have certain limitations: (i) it can take several days before steady-state drug concentrations in tissue/plasma are reached (Timmer & Mulders, 2000; Algorta et al., 2017; Liu et al., 2021), with the rate of absorption also affected by menstrual cycle phase (Rock et al., 1947); (ii) with matrix-type rings, drug release rates decrease significantly with time (Malcolm et al., 2016; Boyd et al., 2019); (iii) it is not possible to deliver doses on demand; and (iv) although continuous prolonged drug release may be beneficial for some clinical applications, long-term drug exposure might cause local adverse reactions and tissue irritation (Bounds et al., 1993; Henriksson et al., 1996).

**Figure 1.**
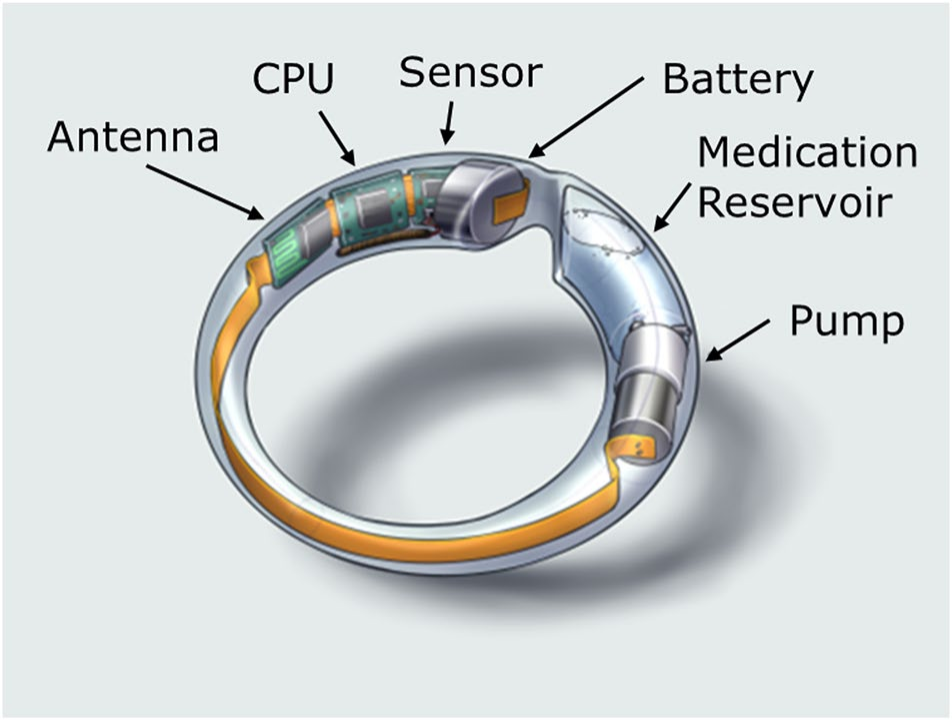
Illustration of the MedRing vaginal ring device.

MedRing was developed to overcome some of the limitations with current vaginal ring technologies, most notably to provide opportunities for (i) administration of liquid doses (e.g. drug solutions, drug suspensions), (ii) on-demand pulsatile drug dosing, and (iii) sampling of vaginal fluid. MedRing is a flexible, user-insertable and removable vaginal ring device, comprising a polyethylene body, a 2.0-mL drug/medication reservoir (which can either be filled with a liquid drug formulation for vaginal administration or used for sampling vaginal fluid), a miniature peristaltic pump, a microprocessor, a battery, various microelectronics, an antenna, a temperature sensor, and a Bluetooth module for communication with an external device (e.g. a smartphone) (Figure 1). MedRing is designed to fold upon pinching for ease of insertion, after which it unfolds to ensure a good fit in the vagina and close contact with the vaginal mucosa. Drug release is controlled by the battery-operated microprocessor, allowing drugs to be released continuously or intermittently at specific time points or monitored and adjusted via a smartphone or computer. The system can be pre-programmed and is able to dispense any liquid-type drug formulation, e.g. solution, suspension, emulsion. The liquid formulation is released through an orifice on the lateral side of the ring, which following insertion remains in intimate contact with the vaginal mucosa. In addition, the MedRing device contains a temperature sensor, potentially permitting the monitoring of patient adherence (Boyd et al., 2015). These adaptations are designed to enable broader therapeutic application compared with conventional vaginal rings and to facilitate administration of drugs that would usually be administered orally.

Available since the 1970s, oxybutynin hydrochloride is a urinary antispasmodic drug that inhibits the muscarinic receptors in the bladder to relieve symptoms associated with overactive bladder, including urinary urgency and frequency (Diokno & Lapides 1972; Electronic Medicines Compendium 2023). The drug is most commonly administered orally using tablet or syrup formulations in doses ranging from 5 to 15 mg (Drugbank 2023). Transdermal patches are also marketed, delivering 3.9 mg per day of oxybutynin (free base form) (Drugbank 2023), and several experimental vaginal formulations, including a conventional silicone elastomer vaginal ring, have been described in the literature (Schröder et al., 2000; Woolfson et al., 2003; Tuğcu-Demiröz et al., 2013; Gittelman et al., 2014). Oxybutynin is rapidly absorbed following oral administration, with 8.2 ng/mL *C*_max_, 0.8 h *t*_max_, and 16 ng/mL h AUC, and an elimination half-life of ∼2 h (Douchamps et al., 1988). Oral oxybutynin is extensively metabolized by CYP3A4 to *N*-desethyloxybutynin in both the liver and the intestine, resulting in a relatively low ∼6% (range 1.6%–10.9%) bioavailability (Douchamps et al., 1988; MacDiarmid 2009). To minimize the hepatic first-pass effect, alternative formulations of oxybutynin have been developed, including oxybutynin-releasing vaginal rings (Woolfson et al., 2003; Kennelly, 2010; Gittelman et al., 2014). Although *N*-desethyloxybutynin-related adverse events (AEs) are reduced with these alternative formulations, local skin reactions are still observed (Davila, 2006; Starkman & Dmochowski, 2006; MacDiarmid, 2009; Gomelsky & Dmochowski, 2012). Intravaginal delivery of oxybutynin using a once-monthly vaginal ring delivering 4 or 6 mg/day demonstrated efficacy and tolerability in a multicenter, 12-week phase 3 trial (Swierzewski et al., 2013). Oxybutynin can cause AEs when taken orally, including dry mouth, constipation, drowsiness, and blurred vision (Yarker et al., 1995; Jirschele & Sand, 2013; Yamada et al., 2018).

Here, we report a first-in-human study using the MedRing device, describing the pharmacokinetics, safety, and tolerability of oxybutynin and its main hepatic metabolite *N*-desethyloxybutynin after intravaginal administration of a single dose of oxybutynin hydrochloride in healthy female subjects.

## Materials and methods

### Study design and participants

This exploratory study was an open-label, single-center study to assess the pharmacokinetics, safety, and tolerability of a single dose of oxybutynin hydrochloride administered in healthy female subjects via the MedRing device (MedRing Alpha 1.0, Demcon Holding, Enschede, The Netherlands). The study was conducted between January 20, 2021 and February 12, 2021.

Subjects included premenopausal and postmenopausal women aged 18–45 years and 50–69 years, respectively. Before study initiation, subjects underwent an initial screening assessment up to 28 days prior to admission and were enrolled in the study if they were assessed to be in general good health, had a body mass index (BMI) of 18–32 kg/m^2^, had a body weight ≥50 kg and, for those of child-bearing potential, were using combination contraceptives containing ethinylestradiol and a progestogen.

Subjects were considered ineligible for the study if (i) they had any clinically significant medical condition or laboratory test results that might complicate the study results; (ii) there were ethical concerns (e.g. a history of sexual abuse); (iii) they had given birth within six months prior to screening; (iv) they had sexual intercourse within 24 h of dosing; (v) they were positive for or at high risk of sexually transmitted diseases; (vi) they used alcohol 24 h prior to screen or study visit; or (vii) they had used a prescription medication or other substance 21 days prior to the study that might influence the study outcomes.

The study was conducted at the Centre for Human Drug Research, Leiden, The Netherlands, in accordance with the Declaration of Helsinki, the International Conference on Harmonisation Good Clinical Practice guidelines, and local and national regulations. The study was approved by the independent medical ethics committee Stichting Beoordeling Ethiek Biomedisch Onderzoek (BEBO), Assen, The Netherlands. The trial was registered with the Netherlands Trial Registry (NL75627.056.20) and EudraCT (2020-0050044-30). All subjects provided written informed consent before any study-specific procedures were performed.

### Clinical study and dosing

The schedule of assessments and study procedures is described in Supplementary Table S1. On Day 1 of the study, a healthcare professional vaginally inserted the MedRing device in subjects at least 15 min prior to dosing. The reservoir of the MedRing vaginal ring device was pre-filled with 2.0 mL of a 100 mg/mL solution of oxybutynin hydrochloride dissolved in a 1:1 vol/vol water/propylene glycol solution.

**Table 1.** Subject demographics and baseline characteristics.

Subject number	Age (years)	Race	Hormonal status	Height (cm)	Weight (kg)	BMI^a^ (kg/m^2^)
1	33	Hispanic	Premenopausal	162.5	60.0	22.7
2	31	White	Premenopausal	170.6	67.3	23.0
3	21	Mixed^b^	Premenopausal	176.9	88.9	28.4
4	21	White	Premenopausal	174.0	67.2	22.2
5	68	White	Postmenopausal	168.6	87.3	30.7
6	69	White	Postmenopausal	167.7	59.7	21.2
7	67	White	Postmenopausal	165.2	69.5	25.3
8	57	White	Postmenopausal	177.0	58.8	18.8

^a^ BMI – body mass index.

^b^ Mixed – white and black mixed race.

The oxybutynin hydrochloride formulation used in the current study was selected following initial preclinical assessment of intravaginal administration of different oxybutynin hydrochloride solutions in Wistar rats. Briefly, eight solution formulations (20.0 mg/mL oxybutynin hydrochloride dissolved in each of the following solvents: 0.9% sodium chloride, isopropyl alcohol, Transcutol^®^ highly purified, dimethylacetamide, *N*-methyl-2-pyrrolidone, dimethyl sulfoxide, polyethylene glycol 400, and propylene glycol) were assessed for biocompatibility and pharmacokinetics (AUC and *C*_max_) following intravaginal administration. Propylene glycol was chosen for further investigation due its high AUC and *C*_max_ values, low viscosity, and good biocompatibility. Subsequently, four formulations of varying water/propylene glycol ratios (100% water, 80:20 water/propylene glycol, 50:50 water/propylene glycol, and 100% propylene glycol) were tested for stability, flow behavior, bioavailability, and preservation. The 50:50 water/propylene glycol solution was determined to be optimal due to good stability (no precipitation) and known preservative properties.

Subjects received a single 30 μL electronically controlled intravaginal dose via the device, equivalent to 3 mg of oxybutynin hydrochloride. Following dosing, MedRing was kept *in situ* for either 2 or 6 h. Subjects were discharged approximately 8 h after drug administration, and an ambulant blood sample was collected the following day (in subjects with MedRing *in situ* for 6 h). Subjects received a follow-up telephone call 5–9 days post-dosing to monitor safety.

Prior to insertion and after removal of MedRing, inspection of the device was performed to ensure quality of construction and dosing accuracy. Pre- and post-dose volume delivery check results are shown in Supplementary Table S2.

**Table 2.** Summary of the pharmacokinetic parameters of oxybutynin and *N*-desethyloxybutynin after a single intravaginal dose of 3 mg oxybutynin hydrochloride using the MedRing vaginal ring device.

Parameter	Unit	*N*	Mean	SD	CV%	GeoMean	GeoCV%	Median	Min.	Max.
Oxybutynin AUC extrapolated	%	6	24.4	13.5	55.3	20.4	84.8	26.2	5.5	45.2
Oxybutynin AUC_8h_	h ng/mL	8	21.4	9.7	45.3	18.5	73.5	22.6	4.5	34.7
Oxybutynin AUC_inf_	h ng/mL	6	34.9	17.4	50.0	29.9	78.7	35.3	8.2	59.8
Oxybutynin CL/F	L/h	6	117.0	108.0	93.0	91.2	78.7	79.0	45.2	334.0
Oxybutynin *C*_max_	ng/mL	8	5.4	2.7	51.0	4.5	77.1	5.6	1.2	9.3
Oxybutynin dose normalized AUC_inf_	h ng/mL/mg	6	12.8	6.4	50.0	11.0	78.7	13.0	3.0	22.0
Oxybutynin dose normalized *C*_max_	ng/mL/mg	8	2.0	1.0	51.0	1.7	77.1	2.1	0.4	3.4
Oxybutynin *t*_1/2_	h	6	8.5	3.5	41.4	7.9	44.2	8.6	4.7	14.3
Oxybutynin *t*_lag_	h	8						0.0	0.0	0.25
Oxybutynin *t*_max_	h	8						2.0	1.0	5.0
Oxybutynin *V*_z/F_	L	6	1391	1294	93.0	1042	92.0	749.0	496.0	3830
*N*-desethyloxybutynin AUC extrapolated	%	7	28.7	16.9	59.1	23.5	87.2	24.9	6.0	52.2
*N*-desethyloxybutynin AUC_8h_	h ng/mL	8	20.7	13.5	65.3	16.4	95.5	18.7	3.3	45.2
*N*-desethyloxybutynin AUC_inf_	h ng/mL	7	51.1	43.1	84.2	34.9	141.6	31.5	5.2	119.0
*N*-desethyloxybutynin *C*_max_	ng/mL	8	3.9	2.5	63.2	3.1	98.4	3.6	0.6	8.1
*N*-desethyloxybutynin *t*_1/2_	h	7	7.7	5.9	76.7	6.38	69.4	5.1	2.9	20.3
*N*-desethyloxybutynin *t*_lag_	h	8						0.4	0.0	0.5
*N*-desethyloxybutynin *t*_max_	h	8						4.0	3.0	5.0
Ratio AUC_22h_*N*-desethyloxybutynin/oxybutynin		4	1.4	0.7	51.9	1.2	50.4	1.1	0.8	2.4
Ratio AUC_8h_*N*-desethyloxybutynin/oxybutynin		8	0.9	0.4	42.5	0.9	34.7	0.8	0.7	1.9
Ratio AUC_inf_ N-desethyloxybutynin/oxybutynin		6	1.3	0.7	57.9	1.1	58.0	0.9	0.6	2.4
Ratio *C*_max_*N*-desethyloxybutynin/oxybutynin		8	0.7	0.3	40.4	0.7	37.1	0.7	0.5	1.4

AUC, area under the curve; AUC_8h_, area under the curve – time from 0 h to 8 h; AUC_22h_, area under the curve – time from 0 h to 22 h; AUC_inf_, area under the curve – time from 0 h to infinity; CL/F, apparent total clearance following extravascular administration; *C*_max_, maximum concentration; CV, coefficient of variation; GeoMean, geometric mean; GeoCV, geometric coefficient of variation; Max., maximum; Min., minimum; SD, standard deviation; *t*_1/2_, terminal elimination half-life; *t*_max_, time to attain *C*_max_; *t*_lag_, absorption half-time; *V*_z_/*F*, apparent volume of distribution during the terminal elimination phase after extravascular administration.

### Pharmacokinetic assessments

The schedule for sampling and pharmacokinetic assessment is provided in Supplementary Table S1. Briefly, blood samples were collected 1 h prior to dosing and at various timepoints post-dose (15, 30, and every 30 min up to 8 h; 20–24 h was also added as a protocol amendment to cover any subjects in whom pharmacokinetic data could not be obtained during prior samplings). Pharmacokinetic parameters included AUC_inf_ (area under the curve – time from 0 to infinity), AUC_8h_ (area under the curve – time from 0 h to 8 h), *C*_max_ (maximum concentration), *t*_1/2_ (terminal elimination half-life), *t*_max_ (time to attain *C*_max_), *t*_lag_ (absorption half-time), CL/F (apparent total clearance following extravascular administration), and *V*_z_/*F* (apparent volume of distribution during the terminal elimination phase after extravascular administration) and were assessed by non-compartmental analysis of plasma concentration over time in R (V4.0.3) using the Perform Pharmacokinetic Non-Compartmental Analysis (PKNCA) package (Denney et al., 2015; The R Core Team, 2020). The parent/metabolite ratio with AUC_(0-tlast)_ (area under the curve – time from 0 to the last measurable concentration) could not be calculated based on the current pharmacokinetic measurement schedule. Plasma oxybutynin concentrations were measured by Ardena Bioanalytical Laboratories (Assen, The Netherlands) using a validated liquid chromatography-mass spectrometry (MS)/MS method. Dose-normalized pharmacokinetic parameters were assessed and included AUC_inf_ and *C*_max_. The parent:metabolite (oxybutynin:*N*-desethyloxybutynin) ratio on the AUC_inf_ was calculated for individuals who had successful linear regression of apparent elimination phases of both oxybutynin and *N*-desethyloxybutynin concentrations.

### Safety and tolerability assessments

The safety and tolerability assessments are detailed in Supplementary Table S1. Anticholinergic adverse effects were assessed by evaluating pupil size, salivary flow, visual near point acuity, and pulse rate. AEs were measured by assessing subjects’ vital signs (pulse rate, systolic blood pressure, diastolic blood pressure), electrocardiogram, physical examination (including a speculum examination), and the need for concomitant medication. Tolerability and comfort of MedRing were evaluated by dichotomous questions asked by the physician or nurse during the study day and upon device insertion and removal.

## Results

### Study population and baseline characteristics

Initially, 23 subjects were screened for inclusion in this study. In total, eight women met the inclusion criteria and completed the study according to protocol (Supplementary Figure S1). Of these, four subjects were premenopausal and four were postmenopausal, aged between 21 and 33 years and 57 and 69 years, respectively. Six subjects were white, one was Hispanic, and one was of mixed race (Table 1). The mean (±standard deviation [SD]) BMI was 24.04 ± 3.65 kg/m^2^.

### Pharmacokinetics of oxybutynin and metabolites

The single 30 µL dose of oxybutynin hydrochloride was administered over a time span of 30 s with the actual delivered dose calculated based upon the pre- and post-calibration volume data for each MedRing device (Supplementary Table S2). The maximum intravariability volume was 4.1 μL, corresponding to a maximum dose of 0.4 mg. For two postmenopausal subjects, a maximum of 20% lower total dosing was calculated.

The mean *C*_max_ of oxybutynin in plasma was 5.4 ± 2.7 ng/mL, observed at a median *t*_max_ 2 h post-dose, with a minimum–maximum of 1–5 h and an estimated mean *t*_1/2_ of 8.5 ± 3.5 h (Table 2). The mean (±SD) *C*_max_ of the active metabolite *N*-desethyloxybutynin reached 3.9 ± 2.5 ng/mL in plasma, observed at a median *t*_max_ 4 h post-dose, with a minimum–maximum of 3–5 h and a mean *t*_1/2_ of 7.7 ± 5.9 h (Table 2).

**Figure 2.**
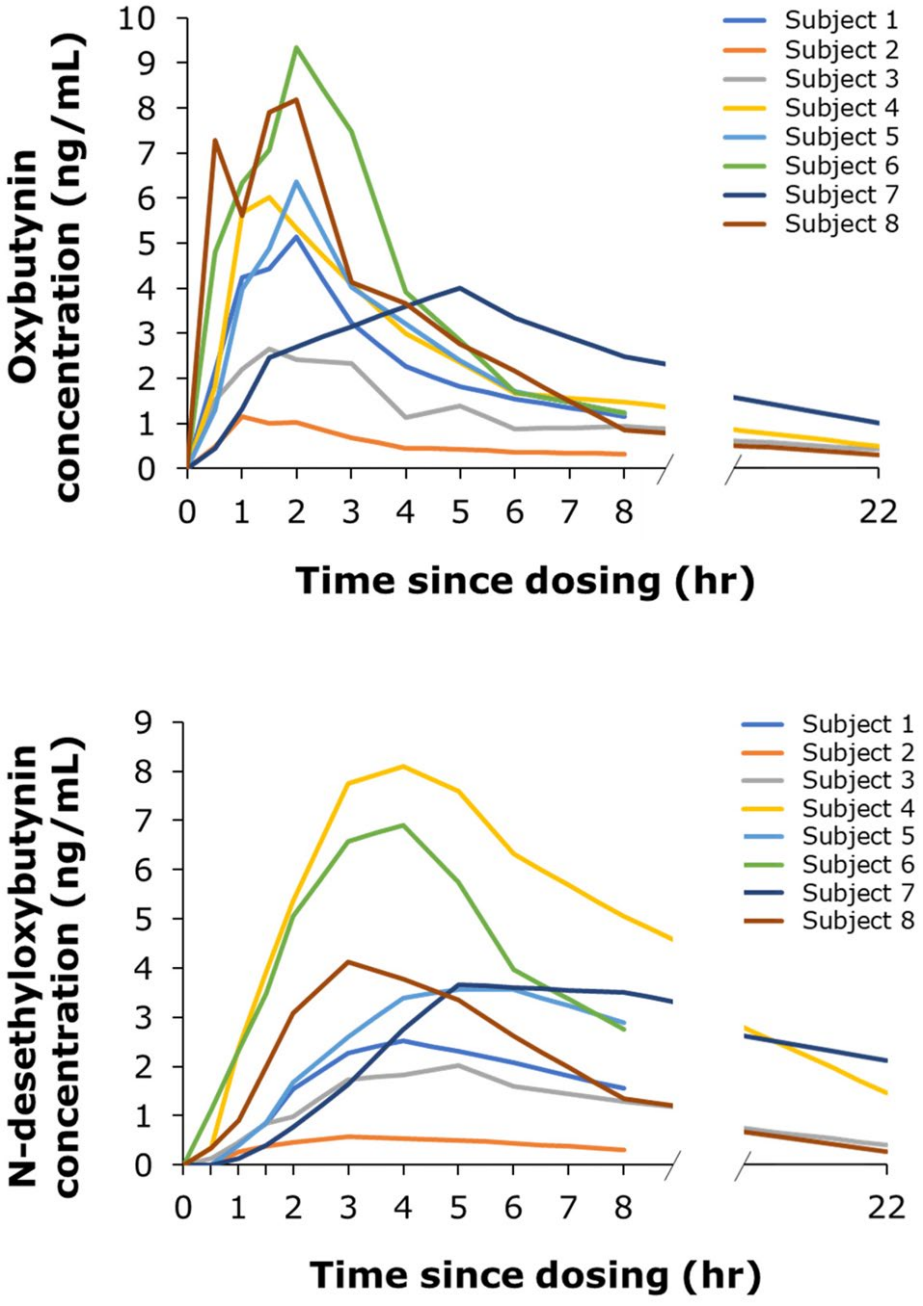
Individual pharmacokinetic profiles of oxybutynin (A) and *N*-desethyloxybutynin (B) up to 22 h after intravaginal dosing with 3 mg oxybutynin hydrochloride.

The observed mean (±SD) AUC_inf_ of oxybutynin and *N*-desethyloxybutynin in plasma was 34.9 ± 17.4 and 51.1 ± 43.1 h ng/mL, respectively. The oxybutynin:*N*-desethyloxybutynin (parent:metabolite) ratio on the AUC_inf_ was 1:1.3, based on data from six subjects who had successful linear regression of apparent elimination phases of both oxybutynin and *N*-desethyloxybutynin concentrations. The individual pharmacokinetic profiles for oxybutynin and *N*-desethyloxybutynin are shown in Figure 2. Semi-logarithmic individual pharmacokinetic profiles are included in Supplemental Figure S2(A,B).

**Figure 3.**
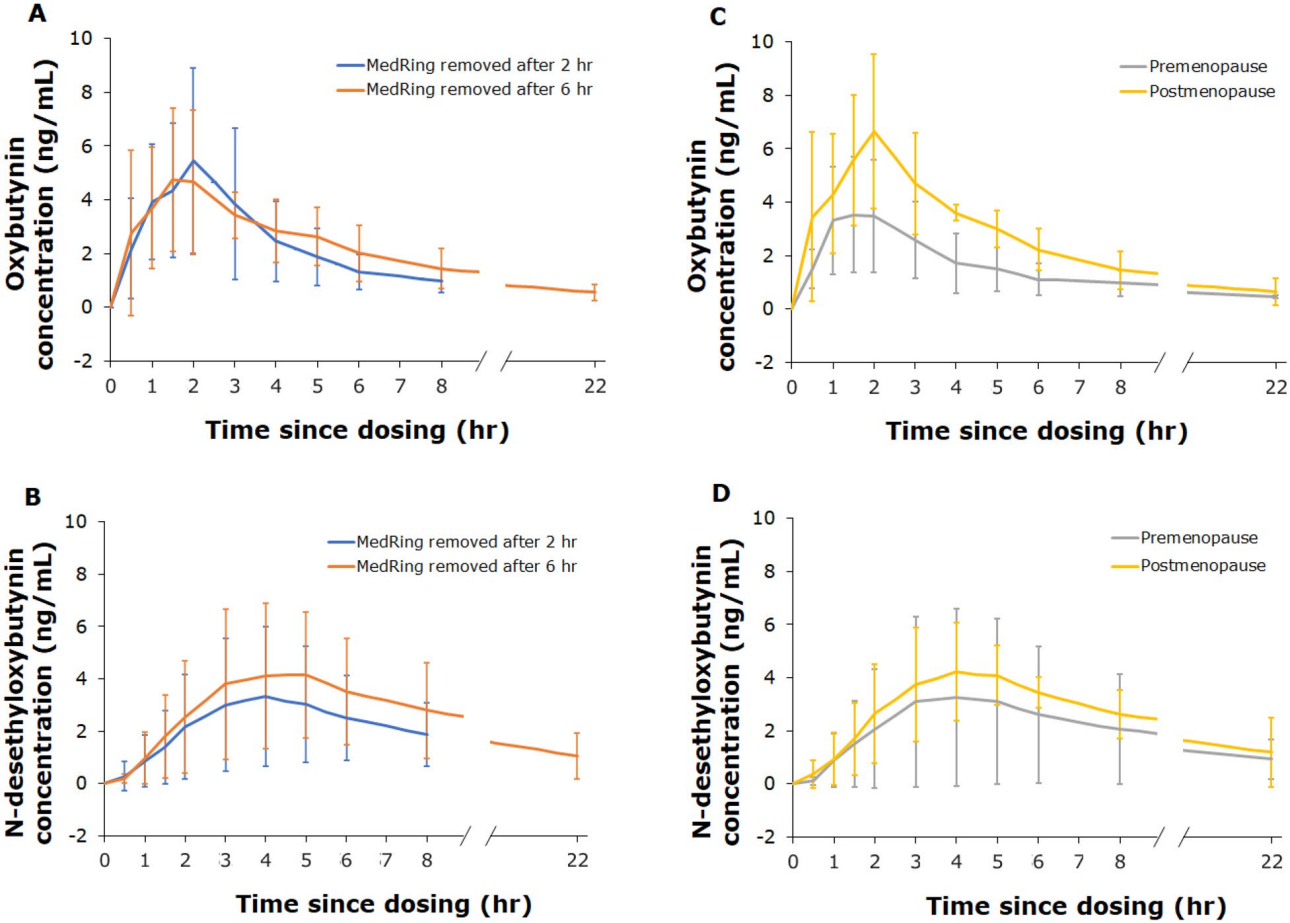
Mean (±SD) pharmacokinetic profiles of oxybutynin and *N*-desethyloxybutynin, 2 h and 6 h (A and B) after removal of MedRing and based on hormonal status (premenopause and postmenopause; C and D) after targeted intravaginal dosing with 3 mg oxybutynin hydrochloride.

There were no apparent differences in the pharmacokinetic profiles of oxybutynin and *N*-desethyloxybutynin depending on whether MedRing was *in situ* for 2 or 6 h (***Figure 3***(A,B)); semi-logarithmic pharmacokinetic profiles are shown in Supplemental Figure S2(C,D). Pharmacokinetic profiles for oxybutynin and *N*-desethyloxybutynin in premenopausal and postmenopausal women were similar (Figure 3(C,D)); semi-logarithmic pharmacokinetic profiles are shown in Supplemental Figure S2(E,F).

### Safety and tolerability

A total of 11 treatment-emergent AEs were reported by five (62.5%) of the eight subjects (Table 3). The reported AEs were mild and transient. There were no serious AEs reported throughout the study. In addition, no local (gynaecological) tolerability issues were reported. Seven (88%) subjects reported not feeling MedRing *in situ* at all and there were no reports of discomfort during insertion or removal (Table 4). No changes suggestive of anticholinergic AEs were observed in salivary flow, vitals, visual near point acuity, or pupil size.

**Table 3.** Treatment-emergent adverse events reported during the study.

	Oxybutynin 100 mg (*n* = 8)	
	Event *n*	Subject *n* (%)
All events	11	5 (62.5)
Eye disorders		
Blurry vision	1	1 (12.5)
Gastrointestinal disorders		
Dry mouth	1	1 (12.5)
Lip dry	1	1 (12.5)
Nausea	1	1 (12.5)
General and administration conditions		
Fatigue	2	2 (25.0)
Feeling hot	1	1 (12.5)
Nervous system disorders (headache)	4	3 (37.5)

**Table 4.** MedRing tolerability assessments through dichotomous questions asked by the physician or the nurse during the study day.

Tolerability question	No (%)	Yes (%)
Was the placement of MedRing uncomfortable?	8 (100%)	0 (0%)
Can you feel the presence of MedRing?	7 (88%)	1 (13%)
Is the presence of MedRing uncomfortable?	8 (100%)	0 (0%)
Can you feel the administration of the medication?	7 (88%)	1 (13%)
Was the removal of MedRing uncomfortable?	8 (100%)	0 (0%)
Did you experience any form of irritation following insertion (2 to 6 hr of wear) and/or removal of MedRing?	8 (100%)	0 (0%)

## Discussion

This first-in-human study demonstrates that the MedRing device was able to safely administer a pre-programmed intravaginal dose of 3 mg oxybutynin hydrochloride in healthy female subjects. The device was well tolerated by all participants throughout the study. Efficacy and tolerability of intravaginal oxybutynin (4 mg and 6 mg vs. placebo vaginal ring) have previously been demonstrated, with dry mouth being the one of the most commonly reported AEs (Swierzewski et al., 2013). Plasma concentration profiles obtained with MedRing were similar to orally administered oxybutynin (typical oral dose 5–15 mg; AUC_inf_ >8 h ng/mL/mg) (Douchamps et al., 1988; Yarker et al., 1995). The plasma concentrations obtained with 3 mg vaginal oxybutynin hydrochloride using MedRing (*C*_max_ 5.4 ng/mL, AUC_8h_ 21.4 h ng/mL) are similar to therapeutic target plasma concentrations achieved after oral and transdermal dosing (target AUC_8h_ ± 25 ng h/mL) (Appell et al., 2003; Zobrist et al., 2003). The plasma oxybutynin levels observed in this study were greater than those previously observed for transdermal gel oxybutynin formulations (AUC 0.8–1.3 h ng/mL/mg) (Dmochowski et al., 2011). In contrast, significantly higher bioavailability of oxybutynin has been reported using intravesical administration (Krause et al., 2013).

The calculated parent:metabolite ratio of 1:1.3 on the AUC_inf_ was comparable to a transdermal patch (with a ratio of 1:1.3) and a transdermal gel (ratio of 1:0.8), and lower than previously observed with immediate (ranging from 1:5 to 1:10) and extended oral release (∼1:4) formulations of oxybutynin (Staskin, 2003; Kennelly, 2010). Orally administered oxybutynin undergoes rapid metabolism in the liver with *N*-desethyloxybutynin as the primary metabolite, resulting in an *N*-desethyloxybutynin:oxybutynin ratio in a range of 5–10 within the circulating blood. The lower metabolite ratio following intravaginal administration compared with oral administration may reduce the incidence of anticholinergic AEs, as *N*-desethyloxybutynin might be largely responsible for oxybutynin-associated anticholinergic side effects. This needs to be assessed in future clinical studies (MacDiarmid, 2009). Some interpatient variability has been observed in the pharmacokinetic analyses of oxybutynin and *N*-desethyloxybutynin, although all patients were within the expected *C*_max_ range, and some interpatient variability is expected with oxybutynin (Yarker et al., 1995; Dmochowski et al., 2011; Gittelman et al., 2014; Kretschmar et al., 2021). However, due to the small number of subjects in our study, it is unclear whether the differences in pharmacokinetics were caused by intersubject variance or dose variance. Intersubject variability may be driven by differences in the size of the vagina, vaginal fluid volume, vaginal pH, and vaginal wall thickness, all of which could affect solubility and absorption of oxybutynin. Dose variance was a potential study limitation as there was no guarantee of consistently delivering the intended 3 mg dose (Supplementary Table S2). A direct comparison of intra- and intersubject variability between oral and intravaginal oxybutynin dosing is warranted in further clinical evaluations.

The results of our study may suggest a higher exposure to oxybutynin in postmenopausal women compared with premenopausal women, although the small number of subjects may limit the validity of this observation. A study of vaginally applied tenofovir gel in premenopausal and postmenopausal women showed that differences in drug absorption were dependent on thickness of the vaginal epithelium, with differences remaining after controlling for vaginal pH and Nugent score (Thurman et al., 2018); similar variations were previously reported, with rate of absorption varying with menstrual cycle phase (Rock et al., 1947). As changes in the vaginal epithelium often occur due to menopause, it will be important to continue assessment of MedRing pharmacokinetics in both premenopausal and postmenopausal women (Flores & Hall, 2022).

No serious AEs were experienced during this study, and fewer treatment-emergent AEs were observed compared with those commonly observed with oral oxybutynin administration (Yarker et al., 1995; Kennelly, 2010). No additional AEs were observed associated with the MedRing device itself, which was well tolerated, and all subjects reported that it was not uncomfortable. In addition, no anticholinergic changes were observed; however, additional clinical evaluation of anticholinergic effects is required in future, larger, placebo-controlled studies. The findings reported here are consistent with other studies that show high acceptance of and tolerability to the vaginal ring format (Gittelman et al., 2014; Duijkers et al., 2020; de Jesus Antunes et al., 2021; Liu et al., 2021).

In the current study, single-dose intravaginal administration of oxybutynin hydrochloride in a solution containing propylene glycol did not result in any significant topical reactions. The oxybutynin hydrochloride formulation used in the study was selected following initial assessment of vaginal administration of different oxybutynin hydrochloride solutions in Wistar rats. Propylene glycol is a well-established excipient used in vaginally administered products, including drug products (U.S. Food & Drug Administration, 2023). Marketed vaginal drug products typically contain propylene glycol quantities in the range 100–200 mg, with daily exposures of propylene glycol as high as 400 mg. In this study, the single 30 μL intravaginal dose of oxybutynin hydrochloride solution administered using MedRing equated to a relatively low ∼15 mg dose of propylene glycol. Furthermore, the potential for mucosal irritation – particularly with multiple dosing regimens – will likely be mitigated by natural *in situ* movements of MedRing due to pelvic muscle activity, thereby avoiding delivery of the microliter doses at the same mucosal location.

Importantly, similar pharmacokinetic profiles were observed when MedRing was *in situ* for 2 or 6 h, indicating that the oxybutynin dose was delivered directly at the time of administration and rapidly absorbed.

The vaginal anatomy and physiology afford opportunities for noninvasive, chronic, local or systemic drug delivery through various formulations and medical devices (Iqbal & Dilnawaz, 2019). Polymeric vaginal rings for the controlled delivery of pharmaceutical compounds have been widely used, particularly as contraceptive devices or for the treatment of local infections, and are continually being improved (Malcolm et al., 2016; Monteiro et al., 2018). The drug release pattern depends upon the design of the device. For example, reservoir-type rings offer controlled drug release at near constant release rates, while matrix-type rings offer relatively large initial release rates followed by declining rates with time. There is currently no option for vaginal pulsatile drug administration or for users to control the timing of drug dosing (apart from the user removing and re-inserting the ring). Furthermore, not all drug compounds can be released effectively by polymeric rings, usually due to relatively high hydrophilicity (resulting in poor solubility in the ring polymer), poor diffusivity (due to large molecular volume of the drug compound), or reaction with the polymeric matrix (Delebecq & Ganachaud, 2012; Dallal Bashi et al., 2019, 2021). The next-generation MedRing vaginal ring has the potential to overcome these limitations.

It will be interesting to further explore the potential of MedRing to deliver medication at a specified time point, with the aim of better individualizing treatment. The potential application of this device to deliver medication dosing during the night and as regularly as needed may be useful when considering chronic conditions that require ongoing medication.

In conclusion, the results of this study demonstrate that intravaginal administration of oxybutynin hydrochloride using the MedRing device has a good safety profile and is well tolerated. MedRing was able to deliver oxybutynin solutions at a prescribed volume without causing irritability in the vaginal mucosa and without causing discomfort during insertion or removal. Thus, MedRing may provide a valuable alternative for the systemic delivery of oxybutynin via the intravaginal route, avoiding AEs by omitting the hepatic first-pass effect and reducing local reactions by controlled, intermittent drug administration instead of continuous drug exposure. Use of the MedRing device for targeted, personalized, intravaginal delivery of systemic medication warrants further investigation in a larger population and with other drug molecules.

## Supplementary Material

Supplemental Material

## Data Availability

The datasets generated during and/or analyzed during the study are available from the corresponding author on reasonable request.
