# Supplementary material for: First-in-human study to assess the pharmacokinetics, tolerability, and safety of single-dose oxybutynin hydrochloride administered via a microprocessor-controlled intravaginal ring
Source: Drug Deliv. 2023 Feb 22;30(1):2180113. doi: 10.1080/10717544.2023.2180113 (PMC9970198; doi:10.1080/10717544.2023.2180113)
Supplement: Supplemental Material [file IDRD_A_2180113_SM9148.zip › Ligalli_Manuscript_Supplemental_table_1.docx]

**Supplementary Table 1.** Schedule of study procedures and assessments.

| Assessment | Screening assessment | Pre-dose | –15 min pre-dose | 0 h | 15 min post-dose | 30 min post-dose | 1 h post-dose | 1.5 h post-dose | 2 h post-dose | 3 h post-dose | 4 h post-dose | 5 h post-dose | 6 h post-dose | 8 h post-dose | 22 (20−24) h post-dose | Telephone call up to 7 days post-dose |
| --- | --- | --- | --- | --- | --- | --- | --- | --- | --- | --- | --- | --- | --- | --- | --- | --- |
| Informed consent | X |  |  |  |  |  |  |  |  |  |  |  |  |  |  |  |
| Demography | X |  |  |  |  |  |  |  |  |  |  |  |  |  |  |  |
| Inclusion and exclusion criteria | X | X |  |  |  |  |  |  |  |  |  |  |  |  |  |  |
| Medical history | X |  |  |  |  |  |  |  |  |  |  |  |  |  |  |  |
| Physical examination | X | X |  |  |  |  |  |  |  |  |  |  |  | X |  |  |
| BMI | X | X |  |  |  |  |  |  |  |  |  |  |  |  |  |  |
| Breath alcohol | X |  |  |  |  |  |  |  |  |  |  |  |  |  |  |  |
| Meals/snack | X |  |  |  |  |  |  |  | X |  | X |  |  | X |  |  |
| Virology | X |  |  |  |  |  |  |  |  |  |  |  |  |  |  |  |
| BsHaem, BsChem, Urinalysis | X | X |  |  |  |  |  |  |  |  |  |  |  |  |  |  |
| UrDrug/Pregnancy testing | X | X |  |  |  |  |  |  |  |  |  |  |  |  |  |  |
| Vaginal smear STD | X |  |  |  |  |  |  |  |  |  |  |  |  |  |  |  |
| Temperature | X | X |  |  |  |  | X |  | X |  | X |  | X |  |  |  |
| ECG | X | X |  |  |  |  |  |  | X |  |  |  |  |  |  |  |
| Symptoms | X | X | X |  | X | X | X | X | X | X | X | X | X | X | X | X |
| Vital signs (HR, BP, RR) | X | X |  |  | X | X | X | X | X | X | X | X | X | X |  |  |
| Anticholinergic adverse effect monitoring: pupil size |  | X |  |  | X | X | X | X | X | X | X | X | X |  |  |  |
| Anticholinergic adverse effect monitoring: near point acuity and salivary flow |  | X |  |  |  |  | X |  | X |  | X |  | X |  |  |  |
| Tolerability question |  |  |  |  | X |  |  |  |  |  |  |  | X | X |  |  |
| (S)AE/concomitant medication | Continuous | | | | | | | | | | | | | | | |
| MedRing insertion |  |  | X |  |  |  |  |  |  |  |  |  |  |  |  |  |
| Oxybutynin administration |  |  |  | X |  |  |  |  |  |  |  |  |  |  |  |  |
| MedRing removal* |  |  |  |  |  |  |  |  | X |  |  |  | X |  |  |  |
| MedRing calibration |  | X |  |  |  |  |  |  | X |  |  |  | X |  |  |  |
| Blood sampling PK |  | X |  |  | X | X | X | X | X | X | X | X | X | X | X |  |
| Discharge |  |  |  |  |  |  |  |  |  |  |  |  |  | X |  |  |

*Removal in half the subjects after 2 hr and the other half after 6 hr.

AE, adverse event; BMI, body mass index; BP, blood pressure; BsHaem, Blood Sample Haematology; BsChem, Blood Sample Chemistry; ECG, electrocardiogram; h, hr(s); HR, heart rate; min, minutes; PK, pharmacokinetic; RR, respiratory rate; SAE, serious AE; STD, sexually transmitted disease; UrDrug, Urine Drug Screen.
