# Supplementary material for: First-in-human study to assess the pharmacokinetics, tolerability, and safety of single-dose oxybutynin hydrochloride administered via a microprocessor-controlled intravaginal ring
Source: Drug Deliv. 2023 Feb 22;30(1):2180113. doi: 10.1080/10717544.2023.2180113 (PMC9970198; doi:10.1080/10717544.2023.2180113)
Supplement: Supplemental Material [file IDRD_A_2180113_SM9148.zip › Ligalli_Manuscript_Supplemental_table_2.docx]

**Supplementary Table 2.** MedRing individual calibration data pre- and post-dose of 3 mg oxybutynin hydrochloride (30 µL solution).

| **Subject** | **Intended dose (µL)** | **Calibration pre-dose (µL)** | **Calibration post-dose (µL)** | **Pre-dose % deviation 30 µL** | **Post dose % deviation 30 µL** | **Total volume MedRing** |
| --- | --- | --- | --- | --- | --- | --- |
| 1 | 30 | 30.3 | 26.2 | 1.1 | -12.6 | 1.41 |
| 2 | 30 | 27.9 | 23.0 | -7.1 | -23.5 | 1.26 |
| 3 | 30 | 28.7 | 26.2 | -4.4 | -12.6 | 1.51 |
| 4 | 30 | 31.2 | 26.2 | 3.8 | -12.6 | 1.59 |
| 5 | 30 | 28.7 | 27.9 | -4.4 | -7.1 | 1.40 |
| 6 | 30 | 30.3 | 27.9 | 1.1 | -7.0 | 1.68 |
| 7 | 30 | 26.2 | 23.8 | -12.6 | -20.8 | 1.84 |
| 8 | 30 | 26.2 | 23.8 | -12.6 | -20.8 | 1.24 |
