# Supplementary figures and images for: First-in-human study to assess the pharmacokinetics, tolerability, and safety of single-dose oxybutynin hydrochloride administered via a microprocessor-controlled intravaginal ring
Source: Drug Deliv. 2023 Feb 22;30(1):2180113. doi: 10.1080/10717544.2023.2180113 (PMC9970198; doi:10.1080/10717544.2023.2180113)

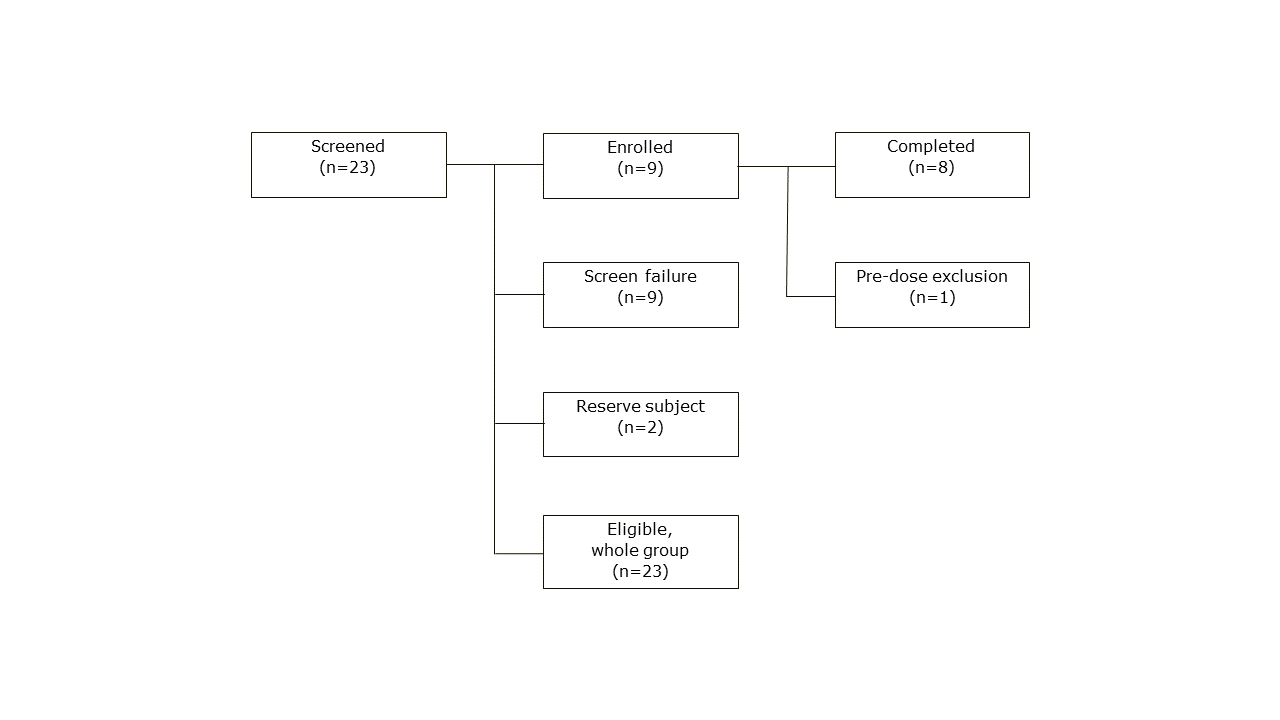

Supplement: Supplemental Material [file IDRD_A_2180113_SM9148.zip › LiGalli_manuscript_Supplemental_figure_1_Subject_disposition.png]

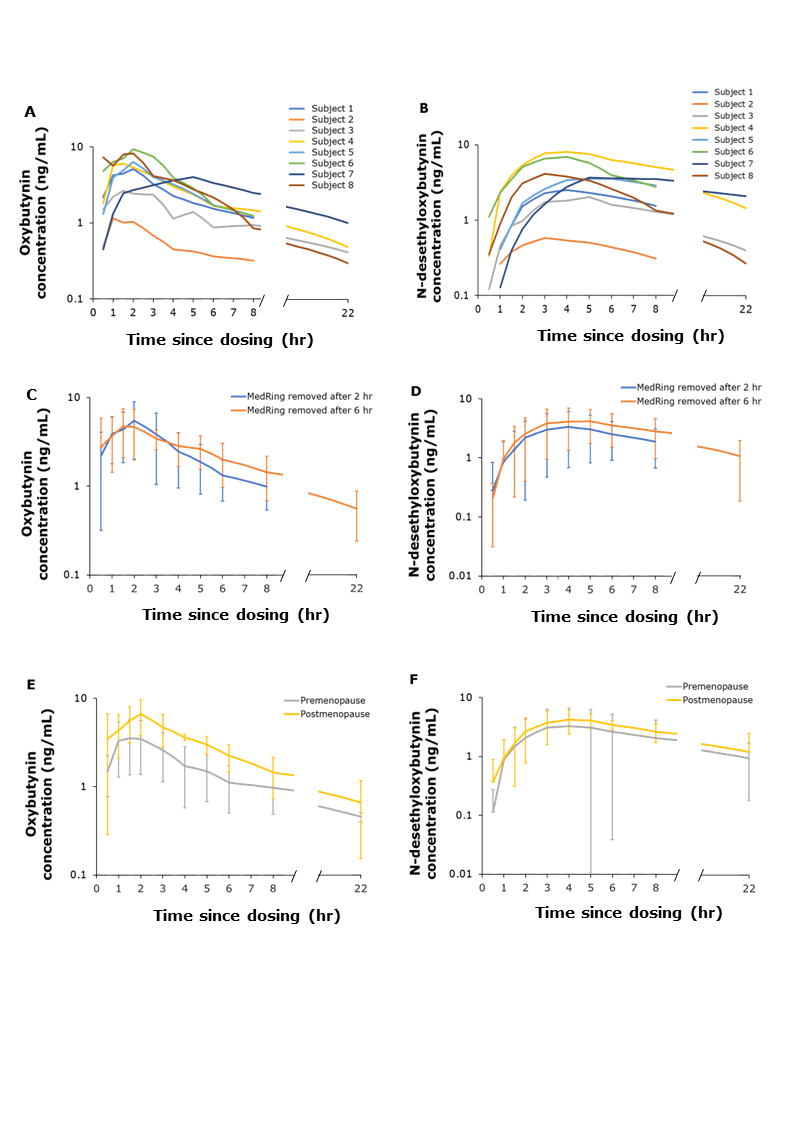

Supplement: Supplemental Material [file IDRD_A_2180113_SM9148.zip › LiGalli_manuscript_Supplemental_figure_2_Semi_logarithmic_scale_pharmacokinetic_profiles.png]
